# Massive pericardial effusion and pleural effusion: cardiac and pleural infiltration in newly diagnosed acute myeloid leukemia

**DOI:** 10.1186/s43046-021-00081-5

**Published:** 2021-09-01

**Authors:** Tao Ma, Hongyun Xing, Xiaofeng Zhu, Pengqiang Wu, Xiaoming Li, Yan Chen

**Affiliations:** Haematology Department, Affiliated Hospital of Southwest Medical University, Luzhou, China

To the Editor:

Acute myeloid leukemia (AML) is a highly heterogeneous hematologic malignancy and is the most common form of acute leukemia in adults [1]. The main clinical manifestations of AML are infection, fever, hemorrhage, and infiltration. The infiltration sites of AML are more common in the spleen, lymph nodes, and central nervous system, while simultaneous infiltration of AML into the cardiac and pleura is less commonly reported. We treated a patient with AML infiltrating both the cardiac and pleura. The patient developed malignant arrhythmia and severe dyspnea, and after we gave the patient percutaneous pericardial drainage and chemotherapy, the patient’s symptoms were relieved and AML was controlled.

A 51-year-old man with more than 10 days of fatigue, dizziness, was admitted to our hospital. Through morphology, immunology, cytogenetics, and molecular biology, he was diagnosed with AML1-ETO leukemia. On admission, the electrocardiogram (ECG) of the patient was normal, and a small pericardial effusion was observed on CT (Fig. 1 (1A)). One week later, there was a significant increase in pericardial effusion and a right pleural effusion (Fig. 1 (1B)). Ten days after admission, the patient had obviously difficult breathing and palpitations, and ECG indicated frequent ventricular premature beat, CT showed massive pericardial effusion and pleural effusion (Fig. 1 (2A, 2B)). Massive pericardial effusion was seen in the bedside echocardiogram, and in the pericardium cavity, a large number of fibrous strips can be seen, some of which were honeycombed. The blood routine indicated that white blood cells were 43 × 10^9^/L, hemoglobin was 62 g/L, and platelets were 21 × 10^9^/L. To relieve the patient’s symptoms, percutaneous pericardial drainage and indwelling catheter were performed, and 500 ml of dark red pericardial effusion was drained. At the same time, HA (homoharringtonine plus cytarabin) regimen was used to control AML. Leukemic cells accounted for 12% on pericardial effusion smear and 6.71% on flow cytometry analysis of pericardial effusion (Fig. 1 (3A, 3B)). From these two tests, we were able to consider that massive pericardial effusion was caused by leukemic cells infiltration of the heart. Flow cytometry analysis of pleural effusion showed that leukemia cells accounted for 6.61% (Fig. 1 (3C)). From this examination, we knew that leukemic pleural infiltration was one of the reasons of pleural effusion. One week after the chemotherapy, the patient’s condition improved and the pericardial puncture tube was planned to be removed (Fig. 1 (3D)). AML achieved complete remission after 1 month of chemotherapy, and pericardial effusion and pleural effusion were significantly reduced (Fig. 1 (4A)). The patient was discharged. Reexamination 1 month after discharge revealed only a small amount of pericardial effusion (Fig. 1 (4B)).

**Fig. 1:**
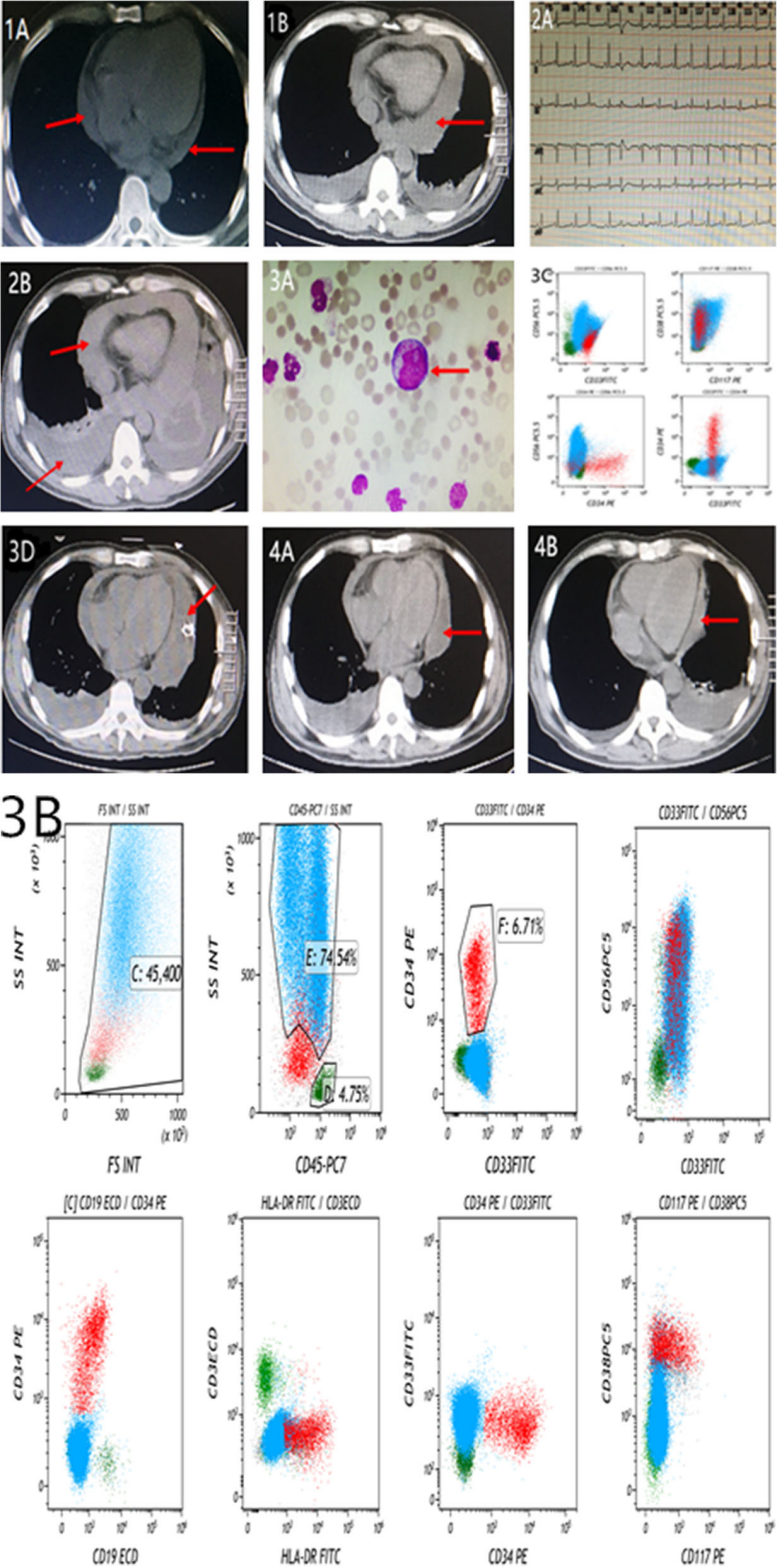
Summary of main laboratory parameters of the patient. **1A** On admission, a small amount of pericardial effusion was found on CT examination. **1B** One week after admission, the patient had a significantly increased pericardial effusion and a right pleural effusion. **2A** Ten days after admittance, ECG suggested frequent premature ventricular contractions. **2B** Ten days after admission, pericardial effusion and pleural effusion further increased. **3A** A smear of pericardial effusion showed leukemic cell. **3B** Leukemic cells were found by flow cytometry analysis of pericardial effusion. **3C** Leukemia cells were found by flow cytometry analysis of pleural effusion. **3D** The patient was placed with a pericardiocentesis drainage tube. **4A** One month after chemotherapy, the patient’s pericardial effusion was significantly reduced. **4B** Reexamination 1 month after discharge revealed only a small amount of pericardial effusion

Acute leukemia infiltrates the heart with myocardial thickening, heart failure, etc. [2, 3]. We reported a patient with simultaneous cardiac and pleural infiltration, characterized by massive pericardial effusion and pleural effusion. Flow cytometry is reliable in the diagnosis of leukemic infiltration with pericardial effusion. The treatment of acute leukemia infiltrates the heart is to reduce the patient’s symptoms as well as to chemotherapy for leukemia.

## Data Availability

Not applicable.

## Abbreviations

AML: Acute myeloid leukemia
ECG: Electrocardiogram
HA: Homoharringtonine plus cytarabin
